# Adjustment for reporting bias in network meta-analysis of antidepressant trials

**DOI:** 10.1186/1471-2288-12-150

**Published:** 2012-09-27

**Authors:** Ludovic Trinquart, Gilles Chatellier, Philippe Ravaud

**Affiliations:** 1 Centre Cochrane Français, Paris, France; 2 Université Paris Descartes - Sorbonne Paris Cité, Paris, France; 3 INSERM U738, Paris, France; 4 Assistance Publique-Hôpitaux de Paris, Hôpital Hôtel-Dieu, Centre d'Epidémiologie Clinique, Paris, France; 5 INSERM CIE 4, Paris, France; 6 Assistance Publique-Hôpitaux de Paris, Hôpital Européen Georges Pompidou, Unité de Recherche Clinique, Paris, France

**Keywords:** Network meta-analysis, Publication bias, Small-study effect

## Abstract

**Background:**

Network meta-analysis (NMA), a generalization of conventional MA, allows for assessing the relative effectiveness of multiple interventions. Reporting bias is a major threat to the validity of MA and NMA. Numerous methods are available to assess the robustness of MA results to reporting bias. We aimed to extend such methods to NMA.

**Methods:**

We introduced 2 adjustment models for Bayesian NMA. First, we extended a meta-regression model that allows the effect size to depend on its standard error. Second, we used a selection model that estimates the propensity of trial results being published and in which trials with lower propensity are weighted up in the NMA model. Both models rely on the assumption that biases are exchangeable across the network. We applied the models to 2 networks of placebo-controlled trials of 12 antidepressants, with 74 trials in the US Food and Drug Administration (FDA) database but only 51 with published results. NMA and adjustment models were used to estimate the effects of the 12 drugs relative to placebo, the 66 effect sizes for all possible pair-wise comparisons between drugs, probabilities of being the best drug and ranking of drugs. We compared the results from the 2 adjustment models applied to published data and NMAs of published data and NMAs of FDA data, considered as representing the totality of the data.

**Results:**

Both adjustment models showed reduced estimated effects for the 12 drugs relative to the placebo as compared with NMA of published data. Pair-wise effect sizes between drugs, probabilities of being the best drug and ranking of drugs were modified. Estimated drug effects relative to the placebo from both adjustment models were corrected (i.e., similar to those from NMA of FDA data) for some drugs but not others, which resulted in differences in pair-wise effect sizes between drugs and ranking.

**Conclusions:**

In this case study, adjustment models showed that NMA of published data was not robust to reporting bias and provided estimates closer to that of NMA of FDA data, although not optimal. The validity of such methods depends on the number of trials in the network and the assumption that conventional MAs in the network share a common mean bias mechanism.

## Background

Network meta-analyses (NMAs) are increasingly being used to evaluate the best intervention among different existing interventions for a specific condition. The essence of the approach is that intervention A is compared with a comparator C, then intervention B with C, and adjusted indirect comparison allows for comparing A and B, despite the lack of any head-to-head randomized trial comparing A and B. An NMA, or multiple-treatments meta-analysis (MA), allows for synthesizing comparative evidence for multiple interventions by combining direct and indirect comparisons [1-3]. The purpose is to estimate effect sizes for all possible pair-wise comparisons of interventions, although some comparisons have no available trial.

Reporting bias is a major threat to the validity of results of conventional systematic reviews or MAs [4,5]. Accounting for reporting biases in NMA is challenging, because unequal availability of findings across the network of evidence may jeopardize NMA validity [6,7]. We previously empirically assessed the impact of reporting bias on the results of NMAs of antidepressant trials and showed that it may bias estimates of treatment efficacy [8].

Numerous methods have been used as sensitivity analyses to assess the robustness of conventional MAs to publication bias and related small-study effects [9-20]. Modeling methods include regression-based approaches and selection models. We extend these approaches to NMAs in the Bayesian framework.

## Methods

First, we extended a meta-regression model of the effect size on its standard error, recently described for MAs [21,22]. In this approach, the regression slope reflects the magnitude of the association of effect size and precision (ie, the “small-study effect”), and the intercept provides an adjusted pooled effect size (ie, the predicted effect size of a trial with infinite precision). Second, we introduced a selection model, which models the probability of a trial being selected and is taken into account with inverse weighting in the NMA. Both adjustment models rely on the assumption that biases are exchangeable across the network, ie, biases, if present, operate in a similar way in trials across the network. Third, we applied these adjustment models to datasets created from US Food and Drug Administration (FDA) reviews of antidepressant trials and from their matching publications. These datasets were shown to differ because of reporting bias [23]. We compared the results of the adjustment models applied to published data and standard NMA for published and for FDA data, the latter considered the reference standard.

### Datasets used

**Figure 1:**
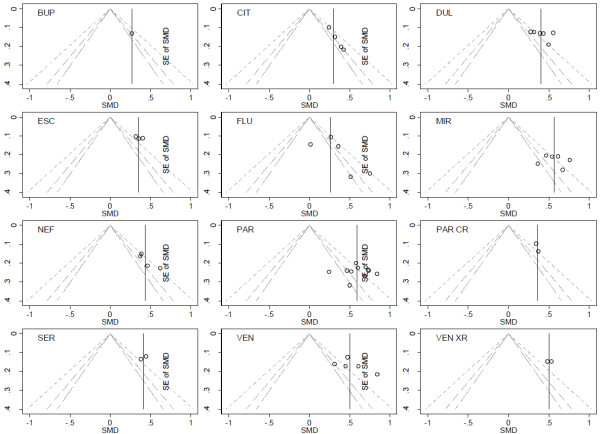
**Contour-enhanced funnel plots forthe antidepressant trials withpublished results.** Each funnel plot is the scatter plot of the treatment effect estimates from individual trials against the associated standard errors; the vertical solid line represents the pooled estimate. In the absence of reporting bias, we might expect a symmetrical funnel plot. We may find the funnel plot is not symmetrical, ie does not resemble an inverted funnel, which may be due to reporting bias, however there are other possible sources of asymmetry. The contour lines represent perceived milestones of statistical significance (long dash p = 0.1; dash p = 0.05; short dash p = 0.01). If studies seem to be missing in areas of non-significance then asymmetry may be due to reporting bias rather than other factors.

A previous review by Turner et al. assessed the selective publication of antidepressant trials [23]. The authors identified all randomized placebo-controlled trials of 12 antidepressant drugs approved by the FDA and then publications matching these trials by searching literature databases and contacting trial sponsors. From the FDA database, the authors identified 74 trials, among which results for 23 trials were unpublished. The proportion of trials with unpublished results varied across drugs, from 0% for fluoxetine and paroxetine CR to 60% and 67% for sertraline and bupropion (Additional file 1: Appendix 1). These entire trials remained unpublished depending on the nature of the results. Moreover, in some journal articles, specific analyses were reported selectively and effect sizes differed from that in FDA reviews. The outcome was the change from baseline to follow-up in depression severity score. The measure of effect was a standardized mean difference (SMD). Separate MAs of FDA data showed decreased efficacy for all drugs as compared to published data, the decrease in effect size ranging from 10% and 11% for fluoxetine and paroxetine CR to 39% and 41% for mirtazapine and nefazodone (Additional file 1: Appendix 1). Figure 1 shows the funnel plots of published data. Visual inspection does not suggest stronger treatment benefit in small trials (ie, funnel plot asymmetry) for any of the 12 comparisons of each drug and placebo.

### Network meta-analysis

The standard model for NMA was formalized by Lu and Ades [2,24,25]. We assume that each trial *i* assessed treatments *j* and *k* among the *T* interventions in the network. Each trial provided an estimated intervention effect size *y*_*ijk*_ of *j* over *k* and its variance *v*_*ijk*_. We assume that *y*_*ijk*_ > 0 indicates superiority of *j* over *k*. Assuming normal likelihood and according to a random-effects model, *y*_*ijk*_ ~ *N*(*θ*_*ijk*_, *v*_*ijk*_) and $\theta_{\text{ijk}}\sim N\left(\Theta_{\text{jk}},\tau^{2}\right)$, where *θ*_*ijk*_ is the true effect underlying each randomized comparison between treatments *j* and *k* and $\Theta_{\text{jk}}$ is the mean of the random-effects effect sizes over randomized comparisons between treatments *j* and *k*. The model assumes homogeneous variance (ie, *τ*_*jk*_^2^ = *τ*^2^). This assumption can be relaxed [2,26]. The model also assumes consistency between direct and indirect evidence: if we consider treatment *b* as the overall network baseline treatment, the treatment effects of *j*, *k*, etc. relative to treatment *b*, $\Theta_{\text{jb}}$, $\Theta_{\text{kb}}$, etc., are considered basic parameters, and the remaining contrasts, the functional parameters, are derived from the consistency equations $\Theta_{\text{jk}}=\Theta_{\text{jb}}-\Theta_{\text{kb}}$ for every *j*, *k ≠ b*.

### Adjustment models

#### Meta-regression model

We used a network meta-regression model extending a regression-based approach for adjusting for small-study effects in conventional MAs [21,22,27-29]. This regression-based approach takes into account a possible small-study effect by allowing the effect size to depend on a measure of its precision. Here, we assume a linear relationship between the effect size and its standard error and the model involves extrapolation beyond the observed data to a hypothetical study of infinite precision. The extended model for NMA is as follows:

$$y_{\text{ijk}}\sim N\left(\gamma_{\text{ijk}},v_{\text{ijk}}\right)$$

$$\gamma_{\text{ijk}}=\theta_{\text{ijk}}+I_{\text{ijk}}\cdot \beta_{\text{jk}}\cdot \sqrt{v_{\text{ijk}}}$$

$$\beta_{\text{jk}}\sim N\left(\beta ,\sigma^{2}\right)$$

$$\theta_{\text{ijk}}\sim N\left(\Theta_{\text{jk}},\tau^{2}\right)$$

$\Theta_{\text{jk}}=\Theta_{\text{jb}}-\Theta_{\text{kb}}$ for every *j*, *k ≠ b*

Figure A in Additional file 2 shows a graphical representation of the model. In the regression equation, *θ*_*ijk*_ is the treatment effect adjusted for small-study effects underlying each randomized comparison between treatments *j* and *k*; *β*_*jk*_ represents the potential small-study effect (ie, the slope associated with funnel plot asymmetry for the randomized comparisons between treatments *j* and *k*). The model assumes that these comparison-specific regression slopes follow a common normal distribution, with mean slope β and common between-slopes variance *σ*^2^. This is equivalent to the assumption that comparison-specific small-study biases are exchangeable within the network. Since we assumed that *y*_*ijk*_ > 0 indicates superiority of *j* over *k*, *β* > 0 would mean an overall tendency for a small-study effect (ie, treatment contrasts tend to be over-estimated in smaller trials). Finally, $I_{\text{ijk}}$ is equal to 1 if a small-study effect is expected to favor treatment *j* over *k*, equal to −1 if a small-study effect is expected to favor treatment *k* over *j*, and equal to 0 when one has no reason to believe that there is bias in either direction (e.g., for equally novel active vs. active treatment). In trials comparing active and inactive treatments (e.g., placebo, no intervention), we can reasonably expect the active treatment to be always favored by small-study bias.

#### Selection model

We use a model that adjusts for publication bias using a weight function to represent the process of selection. The model includes an effect size model (ie, the standard NMA model that specifies what the distributions of the effect size estimates would be with no selection) and a selection model that specifies how these effect size distributions are modified by the process of selection [14,30]. We assume that the probability of selection depends on the standard error of the effect size, as a decreasing function of it. We adopt an approach based on a logistic selection model, as previously used in conventional MAs [18,31].

$$y_{\text{ijk}}\sim N\left(\gamma_{\text{ijk}},v_{\text{ijk}}\right)$$

$$\gamma_{\text{ijk}}=\theta_{\text{ijk}}/w_{i}$$

$$\text{logit}\;w_{i}=\beta_{0jk}+\beta_{1jk}\cdot I_{\text{ijk}}\cdot \sqrt{v_{\text{ijk}}}$$

*β*_0*jk*_ ~ *N*(*β*_0_, *σ*_0_^2^) and *β*_1*jk*_ ~ *N*(*β*_1_, *σ*_1_^2^)

$$\theta_{\text{ijk}}\sim N\left(\Theta_{\text{jk}},\tau^{2}\right)$$

$\Theta_{\text{jk}}=\Theta_{\text{jb}}-\Theta_{\text{kb}}$ for every *j*, *k ≠ b*

Figure B in Additional file 2 shows a graphical representation of the model. In the logistic regression equation, *w*_*i*_ represents the propensity of the trial results to be published, *β*_0*jk*_ sets the overall probability of observing a randomized comparison between treatments *j* and *k*, and *β*_1*jk*_ controls how fast this probability evolves as the standard error increases. We expect *β*_1*jk*_ to be negative, so trial results yielding larger standard errors have lower propensity to be published. The model assumes exchangeability of the *β*_0*jk*_ and *β*_1*jk*_ coefficients within the network. By setting *γ*_*ijk*_ = *θ*_*ijk*_/*w*_*i*_, we define a simple scheme that weights up trial results with lower propensity of being published so that they have a disproportionate influence in the NMA model. *θ*_*ijk*_ is the treatment contrast corrected for the selection process underlying each randomized comparison between treatments *j* and *k*. Finally, $I_{\text{ijk}}$ is defined in the same way as in the preceding section.

### Models estimation

We estimated 4 models: standard NMA model of published data, 2 adjustment models of published data and a standard NMA model of FDA data. In each case, model estimation involved Markov chain Monte Carlo methods with Gibbs sampling. Placebo was chosen as the overall baseline treatment to compare all other treatments. Consequently, the 12 effects of drugs relative to placebo are the basic parameters. For 2 treatments *j* and *k*, *SMD*_*jk*_ > 0 indicate that *j* is superior to *k*. In both the meta-regression and selection models, we assumed that the active treatments would always be favored by small-study bias as compared to placebo; consequently, $I_{\text{ijk}}$ is always equal to 1.

In the standard NMA model, we defined prior distributions for the basic parameters $\Theta_{\text{jb}}$ and the common variance *τ*^2^: $\Theta_{\text{jb}}\sim N\left(0,100^{2}\right)$ and $\tau \sim \text{Uniform}\left(0,10\right)$. In the meta-regression model, we further chose vague priors for the mean slope *β* and common between-slopes variance *σ*^2^: $\beta \sim N\left(0,100^{2}\right)$ and $\sigma \sim \text{Uniform}\left(0,10\right)$. In the selection model, we chose weakly informative priors for the central location and dispersion parameters (*β*_0_, *σ*_0_^2^) and (*β*_1_, *σ*_1_^2^). We considered *p*_*min*_ and *p*_*max*_ the probability of publication when the standard error takes its minimum and maximum values across the network of published data and specified beta priors for these probabilities [32]. The latter was achieved indirectly by specifying prior guesses for the median and 5th or 95th percentile [33]. For trials with standard error equal to the minimum observed value, we assumed that the chances of *p*_*min*_ being < 50% were 5% and the chances of *p*_*min*_ being < 80% were 50%. For trials with standard error equal to the maximum observed value, our guess was that the chances of *p*_*max*_ being < 40% were 50% and the chances of *p*_*max*_ being < 70% were 95%. We discuss these choices further in the Discussion. From this information, we determined Beta(7.52, 2.63) and Beta(3.56, 4.84) as prior distributions for *p*_*min*_ and *p*_*max*_, respectively. Finally, we expressed *β*_0_ and *β*_1_ in terms of *p*_*min*_ and *p*_*max*_ and chose uniform distributions in the range (0,2) on the standard deviations *σ*_0_ and *σ*_1_. For each analysis, we constructed posterior distributions from 2 chains of 500,000 simulations, after convergence achieved from an initial 500,000 simulations for each (burn-in). Analysis involved use of WinBUGS v1.4.3 (Imperial College and MRC, London, UK) to estimate all Bayesian models and R v2.12.2 (R Development Core Team, Vienna, Austria) to summarize inferences and convergence. Codes are reported in the Additional file 1: Appendix 2.

### Models comparison

We compared the results of the 2 adjustment models applied to published data and results of the standard NMA model applied to published data and the FDA data, the latter considered the reference standard. First, we compared posterior means and 95% credibility intervals for the 12 basic parameters and common variance, as well as for the 66 functional parameters (ie, all 12 × 11/2 = 66 possible pair-wise comparisons of the 12 drugs). Second, we compared the rankings of the competing treatments. We assessed the probability that each treatment was best, then second best and third best, etc. We plotted the cumulative probabilities and computed the surface under the cumulative ranking (SUCRA) line for each treatment [34]. Third, to compare the different models applied to published data, we used the posterior mean of the residual deviance and the deviance information criteria [35].

## Results

In the meta-regression model applied to published data, the posterior mean slope *β* was 1.7 (95% credible interval −0.3–3.6), which suggests an overall tendency for a small-study effect in the network. The 12 regression slopes were similar, with posterior means ranging from 1.4 to 1.9. In the selection model applied to published data, the mean slope *β*_1_ was −10.0 (−18.0 – -2.50), so trials yielding larger standard errors tended overall to have lower propensity to be published. In both models, all estimates were subject to large uncertainty (Additional file 1: Appendix 3).

**Figure 2:**
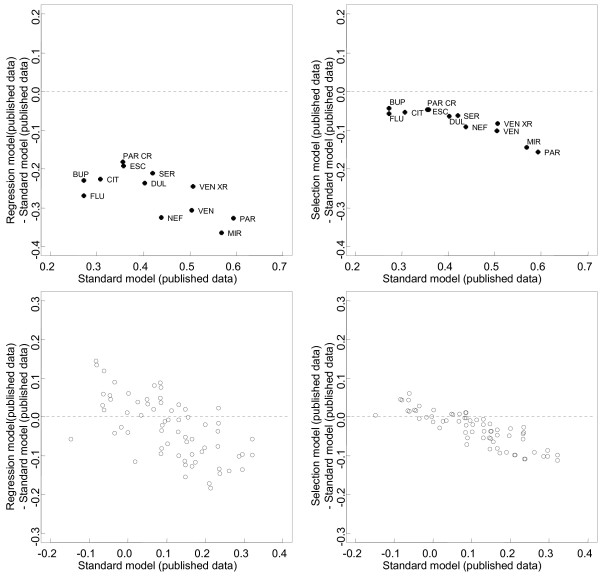
**Difference plots of estimatesof pair-wise comparisons ofthe 12 antidepressant agentsand placebo: regression modelof published data vs.standard network meta-analysis (NMA)model of published data(left panel); selection modelsof published vs. standardNMA model of publisheddata (right panel).** The x-axes show the estimates from the standard NMA model applied to published data, the y-axes show the differences between the estimates from the adjustment (regression or selection) model of published data and the estimates from the standard NMA model of published data. Black dots are the 12 estimated drug effects relative to placebo; white dots are the 66 possible pair-wise comparisons between the 12 drugs.

Table 1 shows the estimates of the 12 basic parameters between each drug and placebo according to the 4 models. As compared with the NMA of published data, both adjustment models of published data showed that the whole 12 estimated drug effects relative to placebo were reduced. For the meta-regression model, the decrease in efficacy ranged from 48% for venlafaxine XR to 99% for fluoxetine. For the selection model, the decrease ranged from 13% for escitalopram to 26% for paroxetine. When considering the functional parameters (ie, the 66 possible pair-wise comparisons between drugs), we found differences between the results of adjustment models and the standard NMA model applied to published trials (Figure 2). The median relative difference, in absolute value, between pair-wise effect sizes from the regression model and the standard NMA model was 57.3% (25% – 75% percentile 30.3% – 97.6%); the median relative difference between the selection model and the standard NMA model was 29.2% (15.1% – 46.1%).

**Table 1:** **Comparison of network meta-analysis(NMA)-based estimates between the2 adjustment models appliedto published data andthe standard NMA modelapplied to US Foodand Drug Administration (FDA)data and to publisheddata**

	**FDA data**	**Published data**		
	**Standard NMA model**	**Regression model**	**Selection model**	**Standard NMA model**
	**Mean (SD)**	**Mean (SD)**	**Mean (SD)**	**Mean (SD)**
*Θ*_*BUP*_	0.176 (0.081)	0.043 (0.256)	0.229 (0.121)	0.271 (0.139)
*Θ*_*CIT*_	0.240 (0.074)	0.081 (0.171)	0.254 (0.073)	0.306 (0.076)
*Θ*_*DUL*_	0.300 (0.054)	0.166 (0.190)	0.340 (0.066)	0.402 (0.058)
*Θ*_*ESC*_	0.310 (0.067)	0.165 (0.193)	0.311 (0.070)	0.357 (0.068)
*Θ*_*FLU*_	0.256 (0.081)	0.004 (0.160)	0.215 (0.068)	0.271 (0.074)
*Θ*_*MIR*_	0.351 (0.070)	0.206 (0.331)	0.424 (0.110)	0.567 (0.092)
*Θ*_*NEF*_	0.256 (0.076)	0.112 (0.260)	0.348 (0.094)	0.437 (0.094)
*Θ*_*PAR*_	0.426 (0.063)	0.267 (0.346)	0.438 (0.105)	0.593 (0.078)
*Θ*_*PAR CR*_	0.323 (0.101)	0.174 (0.187)	0.309 (0.083)	0.354 (0.085)
*Θ*_*SER*_	0.252 (0.077)	0.210 (0.231)	0.359 (0.094)	0.419 (0.094)
*Θ*_*VEN*_	0.395 (0.071)	0.199 (0.224)	0.403 (0.092)	0.504 (0.075)
*Θ*_*VEN XR*_	0.398 (0.094)	0.261 (0.273)	0.423 (0.110)	0.506 (0.107)
*τ*	0.060 (0.037)	0.031 (0.024)	0.024 (0.019)	0.032 (0.025)

Data are posterior means and standard deviations of the basic parameters (Θ), the between-trial heterogeneity (τ).

**Figure 3:**
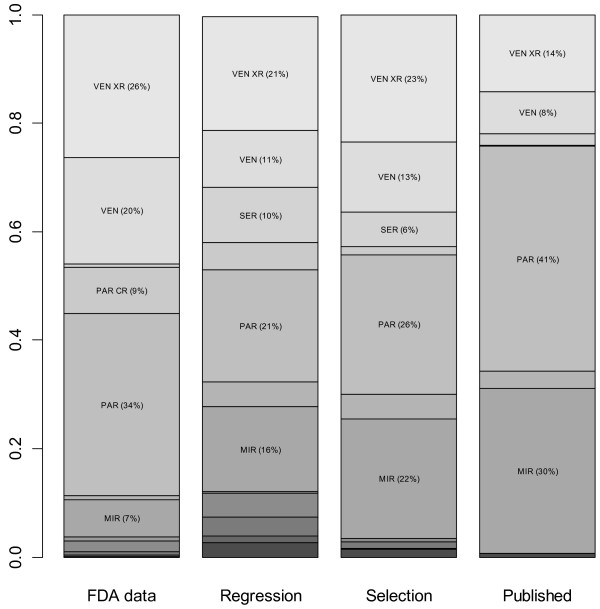
**Probabilities that each antidepressantdrug is the bestaccording to standard NMAof FDA data, regressionmodel, selection model orstandard NMA model ofpublished data.**

Figure 3 summarizes the probabilities of being the best antidepressant. Compared to the standard NMA of published data, adjustment models of published data yielded decreased probabilities of the drug being the best for paroxetine (from 41.5% to 20.7% with the regression model or 25.7% with the selection model) and mirtazapine (from 30.3% to 15.7% or 21.9%). They yielded increased probabilities of the drug being the best for venlafaxine (from 7.9% to 10.6% or 12.8%) and venlafaxine XR (from 14.1% to 21.0% or 23.5%).

**Figure 4:**
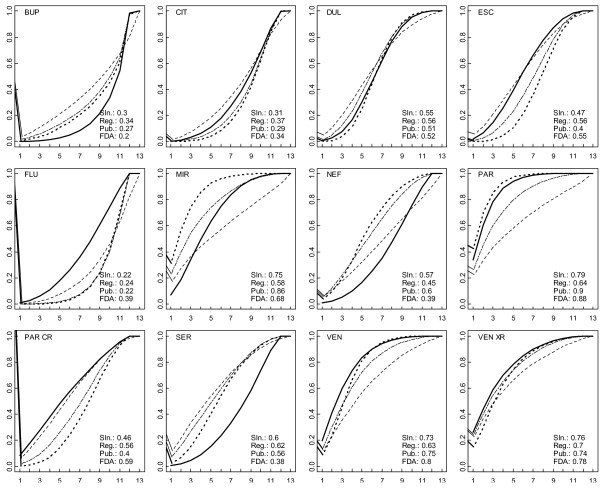
**Cumulative ranking probability plotsfor the 12 antidepressantagents from the standardNMA model applied toFDA data (bold solidline) and published data(bold dotted line) andfrom the 2 adjustmentmodels applied to publisheddata (regression model inplain dashed line andselection model in plaindouble-dashed line).** On each plot, the x-axis shows possible ranks from *r* = 1 up to *r* = 13 and the y-axis shows the cumulative probabilities that the corresponding treatment is among the top *r* treatments. The closer the curve is to the upper left corner, the better the treatment. The surface under the cumulative ranking line is 1 when a treatment is the best and 0 when a treatment is the worst. FDA: standard NMA model applied to FDA data (bold plain line); Pub.: standard NMA model applied to published data (bold dash line); Reg.: regression model applied to published data (dash line); Sln.: selection model applied to published data (long-dash short-dash line).

Figure 4 shows cumulative probability plots and SUCRAs. For the standard NMA of published data, paroxetine and mirtazapine tied for first place and venlafaxine XR and venlafaxine tied for third. The selection model applied to published data yielded a slightly different ranking, with paroxetine, mirtazapine and venlafaxine XR tying for first and venlafaxine was fourth. In the regression model applied to published data, venlafaxine XR was first, venlafaxine and paroxetine tied for second and mirtazapine was fifth.

In adjustment models applied to published data, between-trial heterogeneity and fit were comparable to those obtained with standard NMA of published data (Tables 1 and 2).

**Table 2:** **Comparison of fit andcomplexity between the 2adjustment models and thestandard NMA model, allapplied to published data**

	**Regression model**	**Selection model**	**NMA model**
Mean posterior residual deviance (${\bar{D}}_{\mathrm{res}}$)	31.4	31.5	34.4
Effective number of parameters (pD)	15.9	14.7	13.9
Deviance Information Criterion (DIC)	47.3	46.2	48.3

Lower values of ${\bar{D}}_{\mathrm{res}}$ indicate a better fit to the data. Lower values of the *DIC* indicate a better compromise between model fit and model complexity. A difference in *DIC*s of 5 or more can be considered substantial (http://www.mrc-bsu.cam.ac.uk/bugs/winbugs/dicpage.shtml).

**Figure 5:**
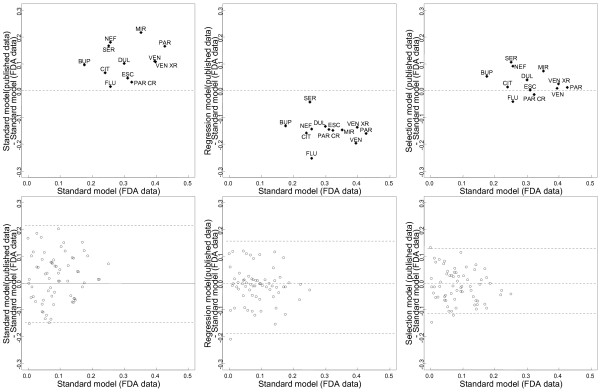
**Difference plots of estimatesof pair-wise comparisons ofthe 12 antidepressant agentsand placebo: standard NMAmodel of published datavs. standard NMA modelof FDA data (upperpanel); regression model ofpublished data vs. standardNMA model of FDAdata (bottom left panel);selection model of publishedvs. standard NMA modelof FDA data (bottomright panel).** The x-axes show the estimates from the standard NMA model applied to FDA data, the y-axes show the differences between the estimates from the adjustment (regression or selection) model of published data and the estimates from the standard NMA model of FDA data. Black dots are the 12 estimated drug effects relative to placebo; white dots are the 66 possible pair-wise comparisons between the 12 drugs.

The estimated drug effects relative to placebo from the regression and selection models were similar to those from the NMA of FDA data for some drugs (Table 1). There were differences when considering the 66 possible pair-wise comparisons between drugs (Figure 5). Results also differed by models regarding the probability of being the best drug and the ranking of drugs. In the standard NMA of FDA data, the probability of being the best drug was 7.3% for mirtazapine, 33.9% for paroxetine, 19.3% for venlafaxine, and 25.7% for venlafaxine XR (Figure 3); paroxetine ranked first, and venlafaxine and venlafaxine XR tied for second (Figure 4).

## Discussion

We extended two adjustment methods for reporting bias from MAs to NMAs. The first method combined NMA and meta-regression models, with effect sizes regressed against their precision. The second one combined the NMA model with a logistic selection model estimating the probability that a trial was published or selected in the network. The former method basically adjusts for funnel plot asymmetry or small study effects, which may arise from causes other than publication bias. The latter adjusts for publication bias (ie, the suppression of an entire trial depending on results). The two models borrow strength from other trials in the network with the assumption that biases operate in a similar way in trials across the domain.

In a specific network of placebo-controlled trials of antidepressants, based on data already described and published previously by Turner et al., comparing the results of adjustment models applied to published data and those of the standard NMA model applied to published data allowed for assessing the robustness of efficacy estimates and ranking to publication bias or related small-study effects. Both models showed a decrease in all basic parameters (ie, the 12 effect sizes of drugs relative to placebo). The 66 contrasts for all possible pair-wise comparisons between drugs, the probabilities of being the best drug and the ranking were modified as well. The NMA of published data was not robust to publication bias and related small-study effects.

This specific dataset offered the opportunity to perform NMAs on both published and FDA data. The latter may be considered "an unbiased (but not the complete) body of evidence" for placebo-controlled trials of antidepressants [28]. The comparison of the results of the 2 models applied to published data and the standard NMA model applied to FDA data showed that the effect sizes of drugs relative to placebo were corrected for some but not all drugs. This observation led to differences in the 66 possible pair-wise comparisons between drugs, the probabilities of being the best drug and the ranking. It suggests that the 2 models should not be considered optimal; that is, the objective is not to produce definitive estimates adjusted for publication bias and related small-study effects but rather to assess the robustness of results to the assumption of bias.

Similar approaches have been used by other authors. Network meta-regression models fitted within a Bayesian framework were previously developed to assess the impact of novelty bias and risk of bias within trials [36,37]. Network meta-regression to assess the impact of small-study effect was specifically used by Dias et al. in a re-analysis of a network of published head-to-head randomized trials of selective serotonin reuptake inhibitors [38]. Along the line of the regression-based approach of Moreno et al. in conventional MA, the authors introduced a measure of study size as a regression variable within the NMA model and identified a mean bias in pair-wise effect sizes. More recently, Moreno et al. used a similar approach to adjust for small-study effects in several conventional MAs of similar interventions and outcomes and illustrated their method using the dataset of Turner et al. [39]. Our approach differed in that we extended this meta-regression approach to NMAs. We used the standard error of treatment effect estimate as the regressor. As well, we specified an additive between-trial variance rather than a multiplicative overdispersion parameter. With the latter, the estimated multiplicative parameter may be < 1, which implies less heterogeneity than would be expected by chance alone. Selection model approaches have been considered recently. Chootrakool et al. introduced an approximated normal model based on empirical log-odds ratio for NMAs within a frequentist framework and applied Copas selection models for some groups of trials in the network selected according to funnel plot asymmetry [40]. Mavridis et al. presented a Bayesian implementation of the Copas selection model extended to NMA and applied their method on the network of Turner et al. [41]. In the Copas selection model, the selection probability depends on both the estimates of the treatment effects and their standard errors. In the extension to NMA, an extra correlation parameter ρ, assumed equal for all comparisons, needs to be estimated. When applied to published data of the network of Turner et al., the selection model we proposed and the treatment-specific selection model of Mavridis et al. yielded close results.

The 2 adjustment models rely on the assumption of exchangeability of selection processes across the network; that is, biases, if present, operate in a similar way in trials across the network. In this case study, all studies were, by construction, industry-sponsored, placebo-controlled trials registered with the FDA, and for all drugs, results of entire studies remained unreported depending on the results [23]. Thus, the assumption of exchangeability of selection processes is plausible. More generally, if we have no information to distinguish different reporting bias mechanisms across the network, an exchangeable prior distribution is plausible, "ignorance implies exchangeability" [42,43]. However, the assumption may not be tenable in other contexts in which reporting biases may affect the network in an unbalanced way. It may operate differently in placebo-controlled and head-to-head trials [44], in older and more recent trials (because of trial registries), and for drug and non-drug interventions [7]. In more complex networks involving head-to-head trials, the 2 adjustment models could be generalized to allow the expected publication bias or small-study bias for active-active trials to differ from that of the expected bias in trials comparing active and inactive treatments [36]. In head-to-head trials, the direction of bias is uncertain but assumptions in defining $I_{\text{ijk}}$ could be that the sponsored treatment is favored (sponsorship bias) [45,46] or that the newest treatment is favored (optimism bias) [37,47,48]. If treatment *j* is the drug provided by the pharmaceutical that sponsored the trial and treatment *k* is not, $I_{\text{ijk}}$ would be equal to 1. Or $I_{\text{ijk}}$ would be equal to 1 if treatment *j* is newer than treatment *k*. However, disentangling the sources of bias operating on direct and indirect evidence would be difficult, especially if reporting bias and inconsistency are twisted together or if the assumed bias directions are in conflict on a loop.

The models we described have limitations. First, they would result in poor estimation of bias and effect sizes when the conventional MAs within the network include small numbers of trials [21]. Second, for the selection model, we specified the weight function. If the underlying assumptions (ie, a logistic link form and the chance of a trial being selected related to standard error) are wrong, the estimated selection model will be wrong. However, alternative weight functions (e.g., probit link) or conditioning (e.g., on the magnitude of effect size) could be considered. Finally, it was implemented with a weakly informative prior, which mainly suggested that the propensity for results to be published may decrease with increasing standard error. There is a risk that prior information overwhelms observed data, especially if the number of trials is low. Although they were somewhat arbitrarily set, our priors for the selection model parameters were in line with the values in previous studies using the Copas selection model [12,49]. Different patterns of selection bias could be tested, for instance, by considering various prior modes for *p*_*min*_ and *p*_*max*_, the probabilities of publication when the standard error takes its minimum and maximum values across the network [15].

## Conclusions

In conclusion, addressing publication bias and related small-study effects in NMAs was feasible in this case study. Validity may be conditioned by sufficient numbers of trials in the network and assuming that conventional MAs constituting the network share a common mean bias. Simulation analyses are required to determine under which condition such adjustment models are valid. Application of such adjustment models should be replicated on more complex networks, ideally representing the totality of the data as in Turner's, but our results confirm that authors and readers should interpret NMAs with caution when reporting bias has not been addressed.

## Competing interest

The authors declared that they have no competing interest.

## Authors' contributions

LT provided substantial contributions to conception and design, analysis and interpretation of data, drafted the article and revised it critically for important intellectual content. GC and PR provided substantial contributions to design and interpretation of data, and revised the article critically for important intellectual content. All authors read and approved the final manuscript.

## Financial disclosure

Grant support was from the French Ministry of Health Programme Hospitalier de Recherche Clinique National (PHRC 2011 MIN01-63) and European Union Seventh Framework Programme (FP7 – HEALTH.2011.4.1-2) under grant agreement n° 285453 (http://www.open-project.eu). The funders had no role in study design, data collection and analysis, decision to publish, or preparation of the manuscript.

## Pre-publication history

The pre-publication history for this paper can be accessed here:

http://www.biomedcentral.com/1471-2288/12/150/prepub

## Supplementary Material

Additional file 1 **Appendix 1.** Summary effect sizes for the 12 comparisons of each antidepressant agent and placebo. **Appendix 2.** Winbugs codes. **Appendix 3.** Estimated parameters in the adjustment models applied to published data. Click here for file

Additional file 2 **Figures.** Graphical representation of the adjustment models (A) regression model and (B) selection model. A solid arrow indicates a stochastic dependence and a hollow arrow indicates a logical function. Click here for file
