# Supplementary material for: Adjustment for reporting bias in network meta-analysis of antidepressant trials
Source: BMC Med Res Methodol. 2012 Sep 27;12:150. doi: 10.1186/1471-2288-12-150 (PMC3537713; doi:10.1186/1471-2288-12-150)
Supplement: Additional file 1 — Appendix 1. Summary effect sizes for the 12 comparisons of each antidepressant agent and placebo. Appendix 2. Winbugs codes. Appendix 3. Estimated parameters in the adjustment models applied to published data. [file 1471-2288-12-150-S1.docx]

**Additional File 1**

Appendix 1: Summary effect sizes for the 12 comparisons of each antidepressant agent and placebo

|  | FDA data | | | Published data | | |
| --- | --- | --- | --- | --- | --- | --- |
| Drug | N | SMD (95%CI) | Τ² | N | SMD (95%CI) | Τ² |
| Bupropion | 3 | 0.17 [0.04; 0.31] | 0.00 | 1 | 0.27 [0.01; 0.53] | NA |
| Citalopram | 5 | 0.25 [0.10; 0.38] | 0.00 | 4 | 0.30 [0.16; 0.44] | 0.00 |
| Duloxetine | 8 | 0.30 [0.21; 0.40] | 0.00 | 6 | 0.40 [0.29; 0.51] | 0.00 |
| Escitalopram | 4 | 0.31 [0.18; 0.44] | 0.00 | 3 | 0.36 [0.23; 0.48] | 0.00 |
| Fluoxetine | 5 | 0.26 [0.06; 0.45] | 0.02 | 5 | 0.29 [0.01; 0.49] | 0.02 |
| Mirtazapine | 10 | 0.35 [0.17; 0.54] | 0.04 | 6 | 0.57 [0.39; 0.75] | 0.00 |
| Nefazodone | 6 | 0.26 [0.12; 0.40] | 0.00 | 4 | 0.44 [0.26; 0.61] | 0.00 |
| Paroxetine | 16 | 0.42 [0.30; 0.54] | 0.00 | 10 | 0.59 [0.44; 0.74] | 0.00 |
| Paroxetine CR | 3 | 0.32 [0.15; 0.49] | 0.00 | 3 | 0.36 [0.20; 0.51] | 0.00 |
| Sertraline | 5 | 0.26 [0.12; 0.39] | 0.00 | 2 | 0.42 [0.24; 0.60] | 0.00 |
| Venlafaxine | 6 | 0.40 [0.24; 0.55] | 0.01 | 5 | 0.51 [0.36; 0.65] | 0.00 |
| Venlafaxine XR | 3 | 0.40 [0.18; 0.62] | 0.02 | 2 | 0.51 [0.30; 0.71] | 0.00 |

Weighted mean effect-size values for each drug were derived using a random-effects model with the method of DerSimonian and Laird. N: number of trials; SMD (95%CI): summary standardized mean difference of drug vs. placebo derived from random effects meta-analysis (95% confidence interval); Τ² (SE): between-trial variance as a measure of heterogeneity in meta-analysis (standard error); NA: not assessable.

Appendix 2: Winbugs codes

Observed data are *y*, the study-specific SMD, with standard error *se*. In both the meta-regression and selection models, we assumed that the active treatments would always be favored by small-study bias as compared to placebo; consequently, $\mathbb{I}_{ijk}$ is always equal to 1.

| Regression model |
| --- |
| for (i in 1:50) {  w[i]<-1/(se[i]*se[i])  y[i]~dnorm(delta.crude[i],w[i])  delta.crude[i]<-delta[i] + beta[t[i]]*se[i]  delta[i]~dnorm(mean[i],precision)  mean[i] <- d[t[i]] - d[b[i]]  }    d[1]<- 0  for (k in 2:13) { d[k] ~ dnorm(0,0.0001)  beta[k]~dnorm(beta.mean,precBeta)  }  beta.mean~dnorm(0,0.0001)  sd~dunif(0,10)  precision <- 1/(sd*sd)  sdBeta~dunif(0,10)  precBeta <- 1/(sdBeta*sdBeta) |
| Selection model |
| for (i in 1:50) { w[i]<-1/(se[i]*se[i])  y[i]~dnorm(delta.crude[i],w[i])  delta.crude[i]<-delta[i]/weight[i]  logit(weight[i])<-beta0[t[i]]+beta1[t[i]]*se[i]  delta[i]~dnorm(mean[i],precision)  mean[i]<- d[t[i]] - d[b[i]]  }    d[1]<- 0  for (k in 2:13) { d[k]~dnorm(0,0.0001)  beta0[k]~dnorm(beta0.mean,precBeta0)  beta1[k]~dnorm(beta1.mean,precBeta1)  }    sd~dunif(0,10)  precision<-1/(sd*sd)  p0~dbeta(3.56,4.84)  p1~dbeta(7.52,2.63)  beta0.mean<-(logit(p1)/0.098-logit(p0)/0.3180)/(1/0.098-1/0.3180)  beta1.mean<-(logit(p1)-logit(p0))/(0.098-0.3180)  sdBeta0~dunif(0,2)  sdBeta1~dunif(0,2)  precBeta0<-1/(sdBeta0*sdBeta0)  precBeta1<-1/(sdBeta1*sdBeta1) |

Appendix 3: Estimated parameters in the adjustment models applied to published data

| Regression model | |  | Selection model | | | |
| --- | --- | --- | --- | --- | --- | --- |
| *β_BUP_* | 1.713 (1.637) |  | *β_0BUP_* | 3.360 (1.311) | *β_1BUP_* | -10.002 (4.143) |
| *β_CIT_* | 1.618 (1.097) |  | *β_0CIT_* | 3.345 (1.299) | *β_1CIT_* | -10.022 (4.128) |
| *β_DUL_* | 1.77 (1.358) |  | *β_0DUL_* | 3.346 (1.300) | *β_1DUL_* | -10.011 (4.140) |
| *β_ESC_* | 1.738 (1.648) |  | *β_0ESC_* | 3.361 (1.312) | *β_1ESC_* | -10.013 (4.138) |
| *β_FLU_* | 1.776 (0.944) |  | *β_0FLU_* | 3.263 (1.284) | *β_1FLU_* | -10.098 (4.083) |
| *β_MIR_* | 1.623 (1.41) |  | *β_0MIR_* | 3.521 (1.353) | *β_1MIR_* | -9.883 (4.093) |
| *β_NEF_* | 1.804 (1.351) |  | *β_0NEF_* | 3.389 (1.284) | *β_1NEF_* | -10.003 (4.135) |
| *β_PAR_* | 1.354 (1.392) |  | *β_0PAR_* | 3.659 (1.411) | *β_1PAR_* | -9.747 (4.052) |
| *β_PAR CR_* | 1.612 (1.482) |  | *β_0PAR CR_* | 3.369 (1.306) | *β_1PAR CR_* | -10.008 (4.144) |
| *β_SER_* | 1.637 (1.669) |  | *β_0SER_* | 3.374 (1.308) | *β_1SER_* | -10.001 (4.141) |
| *β_VEN_* | 1.916 (1.319) |  | *β_0VEN_* | 3.252 (1.289) | *β_1VEN_* | -10.087 (4.143) |
| *β_VEN XR_* | 1.671 (1.702) |  | *β_0VEN XR_* | 3.373 (1.317) | *β_1VEN XR_* | -9.996 (4.137) |
| *β* | 1.684 (0.983) |  | *β_0_* | 3.237 (1.043) | *β_1_* | -10.029 (3.977) |
| *σ* | 1.038 (0.916) |  | *σ_0_* | 0.731 (0.525) | *σ_1_* | 0.983 (0.574) |
