# Supplementary material for: Adjustment for reporting bias in network meta-analysis of antidepressant trials
Source: BMC Med Res Methodol. 2012 Sep 27;12:150. doi: 10.1186/1471-2288-12-150 (PMC3537713; doi:10.1186/1471-2288-12-150)
Supplement: Additional file 2 — Figures. Graphical representation of the adjustment models (A) regression model and (B) selection model. A solid arrow indicates a stochastic dependence and a hollow arrow indicates a logical function. [file 1471-2288-12-150-S2.ppt]

## Slide 1
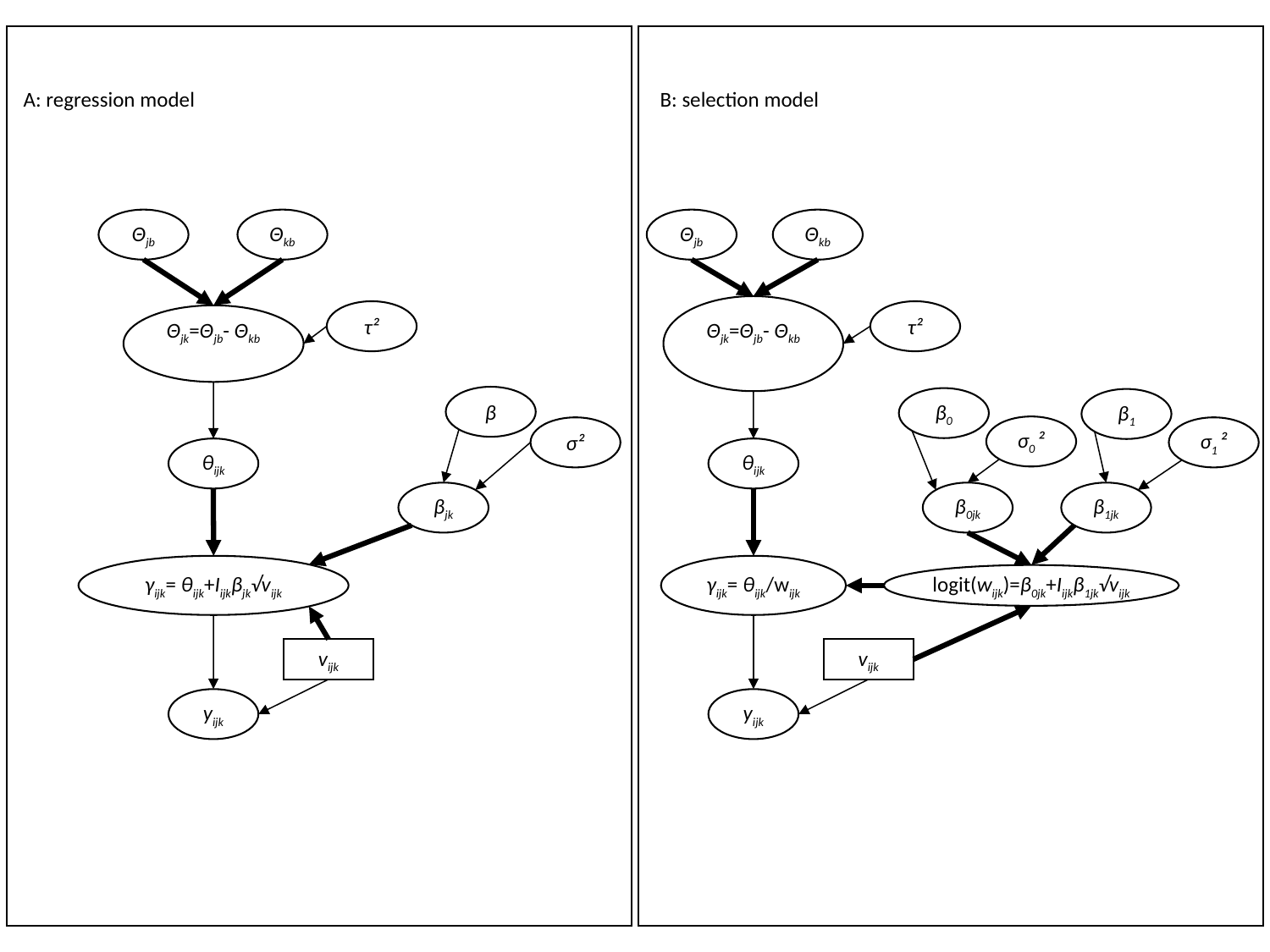

A: regression model
B: selection model
Θjb
Θkb
Θjb
Θkb
Θjk=Θjb- Θkb
τ²
τ²
Θjk=Θjb- Θkb
β
β0
β1
σ0 ²
σ²
σ1 ²
θijk
θijk
βjk
β0jk
β1jk
γijk= θijk+Iijkβjk√vijk
γijk= θijk/wijk
logit(wijk)=β0jk+Iijkβ1jk√vijk
vijk
vijk
yijk
yijk
